# Advancing schizophrenia care: Ongoing Study of a Mobile Application for Personalized Support

**DOI:** 10.1192/j.eurpsy.2024.340

**Published:** 2024-08-27

**Authors:** E. Vass, Á. Szőke, J. Réthelyi, Z. Unoka, L. Hermán, L. Morvai, G. Csukly, G. Strausz, B. Pataki, B. Orbán-Szigeti, S. Blénesi, K. Farkas

**Affiliations:** ^1^Department of Psychiatry and Psychotherapy, Semmelweis University; ^2^Budapest University of Technology and Economics, Budapest, Hungary; ^3^Dept. of Measurement and Information Systems, Budapest University of Technology and Economics; ^4^Faculty of Medicine, Semmelweis University, Budapest, Hungary

## Abstract

**Introduction:**

Psychiatric care faces a significant challenge in the regular monitoring of patient states, predicting relapses, and ensuring treatment adherence. To address this, we aim to develop a mobile application tailored to individual patient needs. This application will revolutionize mental health care by offering real-time monitoring, education, evidence-based interventions, and enhanced communication between patients and clinicians.

**Objectives:**

This ongoing study seeks to develop and evaluate a mobile application for individuals with schizophrenia spectrum disorders, aiming to transform personalized mental health care by addressing critical challenges in psychiatric care.

**Methods:**

The study follows a multi-phase approach, incorporating prototype development, a proof-of-concept trial, and a Randomized Controlled Efficacy Study (RCT). Each phase is informed by iterative stakeholder feedback, ensuring responsiveness to real-world needs and experiences. The research was approved by the Semmelweis University Regional and Institutional Committee of Science and Research Ethics (SE RKEB: 85/2023).

**Results:**

In the pilot phase, we have effectively tracked the daily well-being of participating patients through interactive activities and structured questionnaires. Our experiences in this phase promise to offer valuable insights for the psychiatric community, shedding light on the potential of personalized mental health care interventions.

**Conclusions:**

This ongoing study represents a pivotal step towards redefining interventions for individuals with schizophrenia spectrum disorders. Early results signal a transformative potential in enhancing symptom management. As the study advances, deeper insights will emerge, emphasizing the profound impact of leveraging mobile technology in personalized mental health care.

**Disclosure of Interest:**

None Declared

